# Association between winter cold spells and acute myocardial infarction in Lithuania 2000–2015

**DOI:** 10.1038/s41598-021-96366-9

**Published:** 2021-08-23

**Authors:** Vidmantas Vaičiulis, Jouni J. K. Jaakkola, Ričardas Radišauskas, Abdonas Tamošiūnas, Dalia Lukšienė, Niilo R. I. Ryti

**Affiliations:** 1 Department of Environmental and Occupational Medicine, Lithuanian University of Health Sciences, Tilzes St. 18, 47181 Kaunas, Lithuania; 2 Health Research Institute, Lithuanian University of Health Sciences, Tilzes St. 18, 47181 Kaunas, Lithuania; 3 Faculty of Medicine, Center for Environmental and Respiratory Health Research (CERH), University of Oulu, Oulu, Finland; 4 Biocenter Oulu, University of Oulu, Oulu, Finland; 5 Medical Research Center Oulu, University of Oulu and Oulu University Hospital, Oulu, Finland; 6 Finnish Meteorological Institute, Helsinki, Finland; 7 Laboratory of Population Studies, Institute of Cardiology, Lithuanian University of Health Sciences, Sukileliu St. 15, 50103 Kaunas, Lithuania; 8 Department of Preventive Medicine, Lithuanian University of Health Sciences, Tilzes St. 18, 47181 Kaunas, Lithuania

**Keywords:** Climate sciences, Environmental sciences, Cardiology, Public health

## Abstract

Acute myocardial infarction (AMI) is a major public health problem. Cold winter weather increases the risk of AMI, but factors influencing susceptibility are poorly known. We conducted an individual-level case-crossover study of the associations between winter cold spells and the risk of AMI, with special focus on survival at 28 days and effect modification by age and sex. All 16,071 adult cases of AMI among the residents of the city of Kaunas in Lithuania in 2000–2015 were included in the study. Cold weather was statistically defined using the 5th percentile of frequency distribution of daily mean temperatures over the winter months. According to conditional logistic regression controlling for time-varying and time-invariant confounders, each additional cold spell day during the week preceding AMI increased the risk of AMI by 5% (95% CI 1–9%). For nonfatal and fatal cases, the risk increase per each additional cold spell day was 5% (95% CI 1–9%) and 6% (95% CI − 2–13%), respectively. The effect estimate was greater for men (OR 1.07, 95% CI 1.02–1.12) than for women (OR 1.02, 95% CI 0.97–1.08), but there was no evidence of effect modification by age. Evidence on factors increasing susceptibility is critical for targeted cold weather planning.

## Introduction

Cardiovascular diseases (CVD) remain the leading cause of disease burden in the world. Alarmingly, age-standardized CVD rates have begun to rise in many locations where they were previously declining^1^. 38% of CVD deaths in women and 44% in men are due to acute myocardial infarction (AMI), which constitutes a major public health problem^2^.

The incidence of AMI varies by season, and seems to be highest in the winter^3^. There is previous epidemiological evidence of an association between cold outdoor temperature and AMI^4^, which has been one of the motivators for cold weather plans^5^. In addition, specific activities such as strenuous exercise in a cold environment, e.g. snow shoveling, have been recognized as risk factors of AMI by organizations such as the American Heart Association^6^.

The physiological and pathological effects of short-term exposure to cold are relatively well known^7^. Cold exposure evokes immediate cardiovascular responses via activation of the autonomic nervous system, promoting a mismatch between myocardial oxygen demand and myocardial oxygen supply^8^. In addition, some experimental studies show that short-term cold exposure can induce a pro-thrombotic state within a matter of hours^9^.

Previous attempts have been made to elaborate whether age or sex modify the effect of cold weather on AMI. Such effect modification has been seen in the associations between cold weather and cardiovascular mortality, but the evidence on AMI remains inconclusive^4,10–12^. In addition, only a handful of studies have investigated whether cold weather increases the risk of fatal AMI or nonfatal AMI, and this evidence is inconclusive too^13–15^. Factors modifying the effect of cold spells on AMI can’t be extrapolated from studies on cold weather and cardiovascular mortality or other cardiovascular outcomes, since different diseases have their own characteristics, pathological processes, and risk factors. Furthermore, findings from previous studies on cold weather and AMI can’t be generalized to all other populations or extrapolated to all other climates, warranting more investigations in both local and global settings. Identifying susceptible groups would be important for planning targeted prevention strategies, and could benefit patients through focused clinical and public health activities alike^5^. In addition, assessment of the different outcomes of AMI during cold weather could contribute to a bigger picture of the public health problem.

The general objective of this study was to quantify the associations between cold winter weather and AMI in the general population. The specific objectives were to identify susceptible groups i.e. to assess effect modification by age and sex, and to elaborate whether cold weather is more strongly associated with fatal than nonfatal AMI. An individual-level case-crossover study was conducted to answer the following a priori study questions: (a) do winter cold spells increase the risk of nonfatal AMI, fatal AMI, or both; (b) is the association between cold spells and AMI stronger in men than in women; (c) is the association between cold spells and AMI stronger in the elderly than in the young adults?

## Results

A total of 16,071 adult cases of AMI were recorded in Kaunas during the study years of 2000–2015. 4165 of these occurred during the winter months. 3142 (75.4%) of these experienced nonfatal AMI, while 1023 cases (24.6%) experienced fatal AMI, based on survival at 28 days after the onset of symptoms. Case-fatality rate did not change substantially over the study period 2000–2015. Table 1 shows the characteristics of the study population.

**Table 1:** Characteristics of the study population of all consecutive AMI cases in Kaunas during the study years 2000–2015, by type of AMI, age, sex and time period.

Characteristic	Winter, n (%)	All, n (%)
All cases	4165 (100)	16,071 (100)
Nonfatal AMI	3142 (75.4)	12,329 (76.7)
Fatal AMI	1023 (24.6)	3742 (23.3)
Men	2435 (100)	9421 (100)
Nonfatal AMI	1760 (72.3)	6941 (73.7)
Fatal AMI	675 (27.7)	2480 (26.3)
Women	1730 (100)	6650 (100)
Nonfatal AMI	1382 (79.9)	5388 (81.0)
Fatal AMI	348 (20.1)	1262 (19.0)
25–64 Years	2032 (100)	7800 (100)
Non-fatal AMI	1423 (70.0)	5588 (71.6)
Fatal AMI	609 (30.0)	2212 (28.4)
≥ 65 years	2133 (100)	8271 (100)
Nonfatal AMI	1719 (80.6)	6741 (81.5)
Fatal AMI	414 (19.4)	1530 (18.5)
2000–2003	1032 (100)	4089 (100)
Nonfatal AMI	793 (76.8)	3147 (77.0)
Fatal AMI	239 (23.2)	942 (23.0)
2004–2007	1162 (100)	4443 (100)
Nonfatal AMI	878 (75.6)	3411 (76.8)
Fatal AMI	284 (24.4)	1032 (23.2)
2008–2011	1045 (100)	4036 (100)
Nonfatal AMI	789 (75.5)	3116 (77.2)
Fatal AMI	256 (24.5)	920 (22.8)
2012–2015	926 (100)	3503 (100)
Nonfatal AMI	682 (73.7)	2655 (75.8)
Fatal AMI	244 (26.4)	848 (24.2)

Table 2 shows the descriptive statistics of weather in Kaunas 2000–2015. The 5th percentile threshold for winter cold spells was − 13.9 °C. The 5th percentile threshold of the annual, instead of winter, frequency distribution of daily temperatures used in sensitivity analyses was − 7.6 °C.

**Table 2:** Descriptive statistics of outdoor temperature (T) in Kaunas during the study years 2000–2015.

	Winter	All
Mean of daily T_average_ (SD), °C	− 2.49 (5.81)	7.66 (8.90)
T_average_ (min, max (range)), °C	− 25.2–10.2 (35.4)	− 25.2–27.1 (52.3)
T _average_ quartiles Q1, Q2, Q3, °C	− 5.7, − 1.0, 1.5	1.2, 7.8, 15.0
5th percentile threshold T_average_, °C	− 13.9	− 7.6
Days with T_average_ < 5th percentile threshold, n (%)	73 (5.1)	293 (5.0)
2000–2003, n (%)	18 (24.7)	57 (19.5)
2004–2007, n (%)	16 (21.9)	78 (26.6)
2008–2011, n (%)	17 (23.3)	87 (29.7)
2012–2015, n (%)	22 (30.1)	71 (24.2)

**Figure 1:**
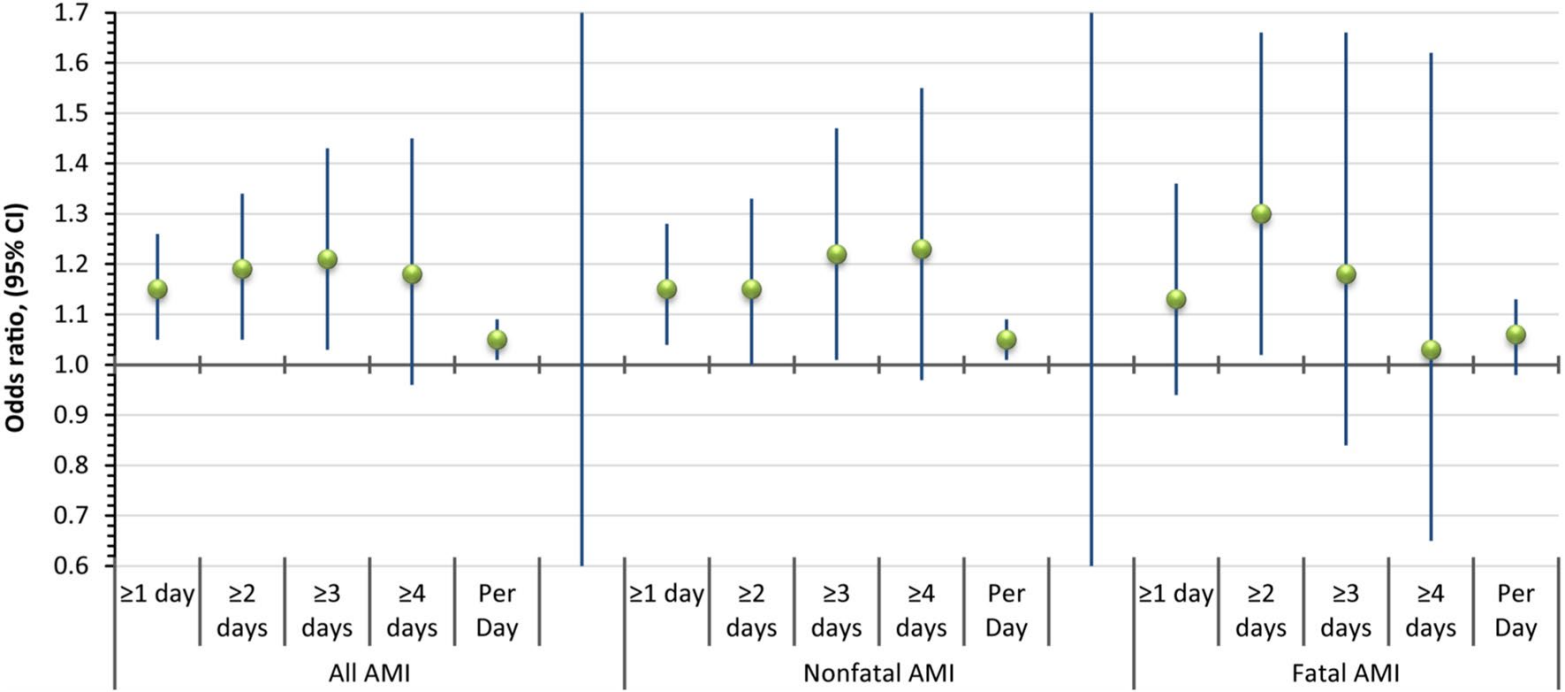
Associations between ≥ 1, ≥ 2, ≥ 3, and ≥ 4 winter cold spell days and AMI in Kaunas, 2000–2015, expressed as odds ratios and 95% confidence intervals.

Based on conditional logistic regression, there was a positive association between winter cold spells and AMI in Kaunas 2000–2015. When using the absolute number of cold spell days during the week preceding AMI as a continuous explanatory variable, each additional cold spell day during the Hazard Period increased the risk of AMI by 5% (odds ratio (OR) 1.05, 95% confidence interval (95% CI) 1.01–1.09). For nonfatal and fatal cases, the risk per each additional cold spell day was 5% (OR 1.05, 95% CI 1.01–1.09) and 6% (OR 1.06, 95% CI 0.98–1.13), respectively. Figure 1 shows the association between ≥ 1, ≥ 2, ≥ 3, and ≥ 4 consecutive winter cold spells days and the risk of AMI, the risk of nonfatal AMI, and the risk of fatal AMI.

Results of the subgroup analyses by sex, age and outcome at 28 days are presented in Table 3. Winter cold spells were associated with an increased risk of AMI in both age groups 25–64 years and ≥ 65 years. There was no difference between the effect estimates, i.e. age did not modify the association between winter cold spells and AMI (Table 3). The association seemed stronger in men (OR 1.07, 95% CI 1.02–1.12) than in women (OR 1.02, 95% CI 0.97–1.08). However, this difference was not statistically significant, z-test 1.32, *p* = 0.18 (Table 3). The subgroup analyses conducted on specific age-sex-strata indicate similar pattern of potential effect modification, i.e. differences between point estimates are greatest by sex, not age (Table 3). In most age-sex-groups, the estimates between all cases, fatal cases, and nonfatal cases are similar. However, two deviations of this pattern are observed, namely in younger women who have higher estimate for fatal AMI and unity estimate for nonfatal AMI, and in older women, who have higher estimate for nonfatal AMI and unity estimate for fatal AMI (Table 3).

**Table 3:** Associations between winter cold spells and AMI by sex and age in Kaunas, 2000–2015, expressed as odds ratios and 95% confidence intervals.

Subgroup	All AMI, OR (95% CI)	Nonfatal AMI, OR (95% CI)	Fatal AMI, OR (95% CI)	z-test (*p*)^b^
Men	1.07 (1.02–1.12)	1.07 (1.02–1.12)	1.08 (0.99–1.17)	*−0.19 (0.85)*
Women	1.02 (0.97–1.08)	1.02 (0.96–1.08)	1.02 (0.90–1.15)	*0 (1)*
* z-test (p)*^*a*^	*1.32 (0.18)*	*1.25 (0.21)*	*0.76 (0.44)*	
25–64 years	1.05 (1.00–1.10)	1.04 (0.98–1.10)	1.07 (0.97–1.17)	*−0.51 (0.61)*
≥ 65 years	1.05 (1.00–1.10)	1.05 (1.00–1.11)	1.04 (0.93–1.16)	*0.15 (0.88)*
* z-test (p)*	*0 (1)*	*−0.24 (0.81)*	*0.38 (0.7)*	
Men 25–64 years	1.06 (1.01–1.13)	1.07 (0.99–1.14)	1.06 (0.96–1.17)	*0.15 (0.88)*
Women 25–64 years	1.01 (0.92–1.11)	0.99 (0.90–1.10)	1.10 (0.86–1.40)	*−0.78 (0.43)*
* z-test (p)*	*0.87 (0.38)*	*1.24 (0.21)*	*−0.28 (0.78)*	
Men ≥ 65 years	1.08 (1.01–1.16)	1.07 (0.99–1.16)	1.13 (0.94–1.36)	*−0.53 (0.6)*
Women ≥ 65 years	1.03 (0.96–1.10)	1.04 (0.96–1.11)	0.99 (0.86–1.15)	*−0.59 (0.55)*
* z-test (p)*	*0.96 (0.33)*	*0.52 (0.6)*	*1.10 (0.27)*	

In addition to testing differences in log odds ratios of the different subgroups within each AMI type were used the z-test and *p* value^a^. The z-test and p-value were also used totest for statistically significant (*p* < 0.05) differences between log odds ratios of nonfatal and fatal AMI in each subgroup.

The findings of the study remained robust under the several sensitivity analyses. Table 4 shows the results of the sensitivity analyses using daily minimum or daily maximum temperature, instead of daily mean temperature, in the definition and assessment of cold spells.

**Table 4:** Results of the sensitivity analyses using daily minimum and maximum, instead of daily mean, temperature in the definition and assessment of cold spells, expressed as odds ratios and 95% confidence intervals.

Exposure	T_min_ cold spells, OR (95% CI)	T_mean_ cold spells, OR (95% CI)	T_max_ Cold spells, OR (95% CI)
≥ 1 day	1.08 (0.99–1.19)	1.15 (1.05–1.26)	1.16 (1.05–1.27)
≥ 2 day	1.18 (1.04–1.34)	1.19 (1.05–1.34)	1.13 (0.99–1.29)
≥ 3 day	1.18 (1.00–1.40)	1.21 (1.03–1.43)	1.26 (1.06–1.51)
≥ 4 day	1.24 (1.01–1.53)	1.18 (0.96–1.45)	1.25 (1.02–1.53)
Per day	1.04 (1.01–1.08)	1.05 (1.01–1.09)	1.05 (1.01–1.09)

When defining the 5th percentile threshold for cold spells from year-round frequency distributions, instead of winter season frequency distributions, of daily temperatures, the effect estimates for all durations of cold spells were smaller and statistically significant, and displayed a similar pattern of an increasing risk of AMI with an increasing duration of cold spell (Table 5). When applying the annual definition of cold spells to the entire data, i.e. not limiting cases or the occurrence of the cold spells into winter months, the estimates remained largely the same (Table 5).

**Table 5:** Results of the sensitivity analyses using different calendar times for the cold spell definition and assessment, expressed as odds ratios and 95% confidence intervals.

Exposure	Winter cold spell, winter cases^a^ OR (95% CI)	Annual cold spell, winter cases^a^ OR (95% CI)	Annual cold spell, all cases^b^ OR (95%CI)
≥ 1 day	1.15 (1.05–1.26)	1.08 (1.00–1.16)	1.06 (0.99–1.13)
≥ 2 day	1.19 (1.05–1.34)	1.09 (1.01–1.18)	1.08 (1.00–1.16)
≥ 3 day	1.21 (1.03–1.43)	1.14 (1.05–1.25)	1.13 (1.03–1.23)
≥ 4 day	1.18 (0.96–1.45)	1.17 (1.06–1.30)	1.15 (1.04–1.26)
Per day	1.05 (1.01–1.09)	1.02 (1.01–1.04)	1.02 (1.00–1.04)

^a^Identically with the main analyses of the study, analysis is limited to cases occurring during the winter months.

^b^Analysis includes cases occurring on all months (n = 16,071).

Sensitivity analyses using detailed 10-year age strata showed no major heterogeneity between the ages 35–94, while the point estimate for those under 35 and those over 94 deviated from the pattern (Table 6).

**Table 6:** Results of the sensitivity analyses using detailed age strata, expressed as odds ratios and 95% confidence intervals.

Age group	OR (95% CI)
25–34	0.82 (0.35–1.94)
35–44	1.06 (0.85–1.31)
45–54	1.03 (0.94–1.12)
55–64	1.06 (1.00–1.13)
65–74	1.03 (0.95–1.11)
75–84	1.06 (0.99–1.14)
85–94	1.06 (0.94–1.19)
95–104	1.39 (0.75–2.57)

Results of the sensitivity analyses of applying the annual cold spell definition to specific age-sex-groups are provided in Table S1 (Supplemental material). Similar with the main analyses, the results indicate greater risk in men than in women, with no effect modification by age, and the associations are generally weaker.

## Discussion

This population-based case-crossover study provided evidence that cold winter weather increases the risk of AMI. Each additional cold spell day during the week preceding AMI increased the risk of AMI approximately 5%. Cold spells seemed to increase the risk of both fatal and nonfatal AMI in similar manner, but random variation in the estimates for fatal AMI makes inference difficult. The effect estimates in both age groups 25–64 and ≥ 65 years were similar. With the exception of the youngest (25–34 years) and oldest (95–104 years), the more detailed stratified analyses by 10-year age groups did not show heterogeneity between strata. The effect estimates were greater among men compared to women overall and in both age groups, although the differences were not statistically significant. The findings of the study remained robust in the several sensitivity analyses.

Previous evidence shows that cold weather increases the risk of AMI^4^, with studies conducted in the different climate regions such as central and western Europe, and Asia^13,14,16,17^. Our findings are in agreement with this evidence and provide additional insights of a similar association in Lithuania, where some previous evidence exists on associations between weather and health^18–20^. However, no studies have comprehensively analyzed the impacts of extremely cold weather for AMI in the Baltic States. Most previous studies have focused on fatal AMI^14,15,21–23^, and fewer studies have used nonfatal AMI as the outcome^14,24^. To our knowledge, there are no studies comparing fatal and nonfatal AMI during cold weather, and such a comparison would be difficult to make in retrospect from separate studies due to comparability issues. Our results are in agreement with previous studies that cold weather increases the risk of both fatal and nonfatal AMI, and provide novel evidence that the risk is similar in both outcomes. This finding is interesting, as it suggests that while environmental circumstances may trigger the ischemic event, they don’t necessarily influence the outcome.

There is some previous evidence that age modifies the effect of cold weather on AMI. Studies conducted in England & Wales, France, China, Norway and Italy have shown that the association between cold weather and AMI is stronger in the elderly than it is in the younger^13,17,25–27^. On one hand, the increased susceptibility in the elderly might be related to impaired thermoregulation^28^. On the other hand, cold-related changes in blood pressure and plasma cholesterol have been reported to be more pronounced in older people, which might contribute towards a greater risk of ischemia and AMI^28,29^. Also, pathways for cold-induced thrombogenesis involve a combination of factors, including hemoconcentration, an inflammatory response, and a tendency for an increased state of hypercoagulability, which may be more pronounced in the elderly^30–33^. In our study, the point estimates for the very young and the very old show a pattern similar with previous studies. However, considering all results together, our interpretation is that there wasn’t evidence of effect modification by age. Disagreement between our results and the previous ones could be related to true differences in the investigated populations and the underlying comorbidities, but differences in methodology can also play a role. The two studies that used methodology most similar to ours, one conducted in China (Beijing) and one in Czech Republic, both reported that the associations between cold weather and AMI hospitalizations and AMI mortality (respectively) were similar among both age groups^15,17^. Considering that most of the previous studies did not perform statistical testing on whether their observed differences could be explained by chance, it seems that the question of effect modification by age remains open.

There are only a handful of studies on effect modification by sex on the topic of cold spells and AMI. 2 previous studies conducted in China and the United States showed that the association between cold spells and AMI was stronger among men than women^17,34^. However, 2 previous studies from England & Wales and Germany show that effect estimates did not differ between men and women^13,14^. In our study, men seemed more susceptible to the effects of cold weather than women, but the difference was not statistically significant. Looking at the patterns of all estimates, this could represent true differences between sexes even though chance variation makes direct inference impossible. There would be several explanations for this finding. Outdoor work is more common among men than women in Lithuania^35^. Men in Lithuania also retire later than women, and may be thus longer exposed to outdoor weather conditions on the way to and from work. Comorbidity profiles are different between sexes^36,37^, and behavioral factors and perception of risk may also differ. This remains a highly valuable future study topic to guide decision making.

This study has several strengths. We applied a homogenous eligibility criteria based on WHO MONICA criteria, and the procedures of case selection did not vary over time. Another strength of our study is the application of the case-crossover design. Case-crossover design is well-suited for studying transient risk factors over time and space^38^, and the current application controls for time-invariant and time-varying confounders. Because we contrast weather during time period A with weather during time period B, the inference is free of inter-individual changes over time. The 7-day hazard period including the day of AMI accommodates both a potential triggering effect and a potential lagged effect of cold weather. Another strength is that we used winter frequency distribution, instead of the more commonly applied annual frequency distribution, of daily temperatures in the definition of cold spells^39^. The sensitivity analyses show that our definition was a stronger predictor of AMI than the old definition, and the findings indicate benefits of preferring seasonal over annual definitions of cold weather in health studies. Another major strength of the study was the formal quantification of heterogeneity between subgroup estimates, which is absent in most studies that investigated effect modification of the association between cold weather and AMI^2,13,14,17,26,27,34^. The fact that our analyses didn’t reveal statistical significance is noteworthy. It seems that there is no quantitative evidence that age or sex modify the effect of cold weather on AMI. Although some qualitative interpretations can be well-justified from the patterns of effect estimates in the context of specific study questions, this notion calls for more studies that specifically and formally focus on this problem. Finally, our extensive sensitivity analyses to assess the robustness of the results could be considered a strength of the study.

A limitation of our study was that even though the Kaunas Ischemic Heart Disease (IHD) Register is prospective in nature, it still captured cases after a time delay. It is possible that some cases occurring in Kaunas 2000–2015 were not captured by the method. However, given the extensive data collection protocol, this number is likely to be so small that it does not influence our results. It is possible that some cases identified and recorded as AMI represented in fact some other pathology than AMI. This is more unlikely in the younger age group (25 to 64 years), because both incident and mortality data in this group were assessed by a combination of symptoms, ECG changes, serum enzyme activity, and autopsy findings. To some extent, diagnostic misclassification is possible in the elderly group aged 65 or more, in which both survivors and non-survivors were recorded according to their clinical diagnosis, without subsequent retrospective verification by the other medical records. This limitation is common to all studies using the WHO MONICA criteria, and its influence on our results is probably not significant. Another limitation of our study was that it is not possible to ascertain whether the AMI cases had been in the area represented by the Kaunas weather station during the week preceding the AMI. Considering that all cases were a) residents of Kaunas, and b) de facto in the catchment area of the relevant hospital just before AMI, bias is highly unlikely. One more limitation of the study is that we didn’t have information on other medical or circumstantial factors which could influence the outcome of AMI. This could be important in cases who underwent cardiopulmonary resuscitation (CPR), for example, because CPR is a procedure with several independent determinants of the outcome. However, CPR and its constituents do not influence the weather, so it shouldn’t be considered a confounder in our statistical setting. The same applies to comorbidities, CCS score, NYHA grade, physical activity, depression, and smoking, which could theoretically be effect modifiers or intermediates, but not confounders. Another limitation of the study is that we did not analyze the role of other meteorological factors such as relative humidity or air pressure. However, in previous studies the role of these factors has been minor compared with temperature^13,40^. Lack of air pollutants in the models should not be considered a limitation of the study. Effects of air pollution on AMI were not controlled, because air pollutants can be seen as intermediate variables in the pathway from cold weather to AMI^41^. Adjusting for the intermediate variables would lead to underestimation of the true effect. This decision was made a priori. Air pollution levels in Kaunas are also relatively low, so their role in mediating the effects of cold spells are likely small in this study^42,43^.

In conclusion, this case-crossover study provided evidence that cold spells increase the risk of AMI. Each additional cold spell day during the week before AMI increased the risk by 5%. The associations seemed equally strong in both fatal and nonfatal AMI. Males seemed more susceptible to the effects of winter cold spells than females, and there was no evidence of effect modification by age. More studies are needed on factors influencing susceptibility to AMI during cold weather, so targeted interventions can become evidence-based.

## Methods

We conducted a case-crossover study of the association between winter cold spells and the risk of AMI in the city of Kaunas, Lithuania, 2000–2015. The study protocol for WHO MONICA study was approved by the Lithuanian Bioethics Committee (ref. No. 14-27/03 December 2001) and the study complies with the Declaration of Helsinki. According to the decision of the Lithuanian Bioethics Committee, consent from subjects was not needed for the study. Prior to analysis, all patient information was anonymized. STROBE guidelines were followed in the reporting.

### Study population

The population-based Kaunas Ischemic Heart Disease (IHD) Register was used to identify cases of AMI for the study. Patients aged at least 25 years, who were permanent residents of the city of Kaunas, and who experienced AMI in Kaunas during the winter months (calendar months December, January, and February) between 2000 and 2015, were included in this study. According to the Lithuanian Statistical Department, the population of the City of Kaunas in the age groups ≥ 25 was 253,154 in the year 2000, and 221,898 in 2015.

### Definition of AMI and the eligibility criteria for the study

The Kaunas Ischemic Heart Disease (IHD) Register has been collected as part of the WHO MONICA project (Monitoring of Cardiovascular Trends and Determinants)^44^. The diagnosis of AMI, and the quality control procedures, are thus based on the WHO MONICA project criteria. Retrospective data collection technique known as “cold pursuit” was used to identify and verify AMI cases^44^. Essentially, for patients 65 years of age and older, AMI was assessed and recorded according to the clinical diagnosis set by the treating physician (ICD-10 I21-I22), whereas in the age group 25–64 years, several additional sources of information were used to identify and confirm the AMI cases, including hospital discharge records, in-hospital documentation, outpatient records, autopsies, and medical-legal records. All suspected AMI events were recorded in special forms translated from the WHO MONICA Project Acute Myocardial Infarction Event Registration Form. The diagnosis of AMI was based on four diagnostic criteria: (1) symptoms of coronary event, (2) dynamic electrocardiogram (ECG) changes indicative of AMI development, (3) changes in serum cardio-specific enzyme activity, and (4) autopsy findings, and a multi-level probability criterion was used for inclusion or exclusion^44^. Multiple attacks of AMI that occurred within 28 days of the onset of first symptoms were considered as a one event. Multiple attacks with more than 28 days apart were considered as separate incidents, and recorded as such. Each case of AMI presented the initial symptoms within the study period. AMI was defined fatal, if death occurred within the first 28 days of its onset. If the patient was alive 28 days after the onset of the attack, AMI was classified nonfatal.

### Exposure assessment

A continuous time-series of daily mean temperatures in the city of Kaunas was obtained from the Lithuanian Hydrometeorological Service under the Ministry of Environment. The data are based on measured values of daily temperatures at the Kaunas monitoring station, located near the local airport (DMS coordinates 54° 53′ 02.7″ N 23° 50′ 09.2″ E). It is the only monitoring station in Kaunas, whose data are supplied for the civil public. This monitoring station corresponds to quality control LST EN ISO 9001:2015 (Certificate No. 9000–493). After manually checking the data for outliers, we formed a frequency distribution of daily mean temperatures over the winter months (December, January, February) of the study period 1.1.2000–31.12.2015. Winter cold spell day was defined as a day with mean temperature below the 5th percentile of the frequency distribution. Specified durations of cold spells were set a priori, with 2 complementary approaches for the main effect: (a) ≥ 1 cold spell day, ≥ 2 cold spell days, ≥ 3 cold spell days, and ≥ 4 cold spell days in a consecutive order during the week preceding AMI, with each stratum analyzed separately; (b) The absolute number of cold spell days during the week preceding AMI as a continuous variable, with values from 0 to 7, without the requirement for consecutive order, including all variations in the one model.

### Statistical analyses

For each case, we defined a one-week Hazard Period preceding the AMI, including the date of AMI and the 6 preceding days, taking into account the potential time lag between the cold exposure and AMI. We used the same calendar days of the other study years to define 15 Reference Periods for each case. The occurrence of cold spells between Hazard Period and the Reference Periods forms a contrast for the statistical analyses. We applied conditional logistic regression using PROC PHREG in SAS, applying the discrete logistic model and forming a stratum for each case. Odds Ratio (OR) with 95% confidence intervals (95% CI) was used as the measure of effect. Long time trends were controlled by an indicator variable of 4-year intervals over the study period. Weekdays and holidays were controlled by the design.

We performed a priori stratified analyses for the different cold spell definitions. We conducted a priori stratified analyses by survival at 28 days. We conducted a priori subgroup analyses by sex and age for 2 age groups: 25–64 and ≥ 65 years. The z-test was used to test for significant difference between log odds ratios with the following Eq. (1):

1 $$ Z = (E_{1} {-}E_{2} )/\sqrt {SE\left( {E_{1} } \right)^{2} + SE\left( {E_{2} } \right)^{2} } $$

where Z denotes the Z-test; *E*_1_ and *E*_2_ are the effect estimates (i.e. ln (RR)) of two subgroups; *SE*(E_1_) and *SE*(E_2_) are corresponding Standard Errors of *E*_1_ and *E*_2_^45^.

Several sensitivity analyses were performed to assess robustness of the results. We replaced our season-specific cold spell definition with the more common annual cold spell definition, where cold spell day is defined as a day with temperature below the 5th percentile of the frequency distribution of daily temperatures over the entire study period, not just winter months. This sensitivity analysis was first performed for the winter cases of AMI, similar to the main analyses, and then for all 16,071 cases of AMI that occurred during the study years regardless of the season. We also repeated the main analyses using T_min_ and T_max_, instead of T_mean_, in the definition and assessment of cold spells. We supplemented the analyses of two age groups with more detailed stratified analyses by 10-year age intervals. The purpose of these analyses was to investigate whether any within-group heterogeneity could have balanced the estimates in a way that could have masked effect modification by age. Finally, we performed the analyses of specific age-sex-groups using both cold spell definitions to ensure these results remain robust (Supplemental Material, Table S1).

All analyses were conducted with SAS (SAS, V.9.4; SAS Institute).

## Supplementary Information

Supplementary Information.

## Data Availability

The data on AMI cases were obtained from Institute of Cardiology under the Lithuanian University of Health Sciences. Data are not accessible online. Temperature data comes from Lithuanian Hydrometeorological Service. Data are not accessible online.
