# Supplementary material for: Association between winter cold spells and acute myocardial infarction in Lithuania 2000–2015
Source: Sci Rep. 2021 Aug 23;11:17062. doi: 10.1038/s41598-021-96366-9 (PMC8382753; doi:10.1038/s41598-021-96366-9)
Supplement: Supplementary file 1 — Supplementary Information. [file 41598_2021_96366_MOESM1_ESM.pdf]

Supplemental Material

Association between winter cold spells and acute myocardial infarction in Lithuania 2000-2015

Vidmantas Vaiciulis, Jouni J.K. Jaakkola, Ričardas Radišauskas, Abdonas Tamošiūnas, Dalia Lukšienė, Niilo R.I. Ryti*

Subgroup	All AMI, OR (95% CI)	Nonfatal AMI, OR (95% CI)	Fatal AMI, OR (95% CI)	z-test (p)[b]
Men	1.03 (1.01-1.06)	1.03 (1.00-1.06)	1.05 (1.01-1.10)	*-0.73 (0.47)*
Women	1.01 (0.98-1.04)	1.01 (0.98-1.04)	1.03 (0.97-1.09)	*-0.58 (0.56)*
z-test (p)[a]	*1.00 (0.31)*	*0.92 (0.36)*	*0.52 (0.6)*	
25-64 years	1.02 (1.00-1.05)	1.01 (0.98-1.04)	1.04 (1.00-1.09)	*-1.10 (0.27)*
≥65 years	1.03 (1.00-1.05)	1.02 (1.00-1.05)	1.04 (0.99-1.10)	*-0.65 (0.51)*
z-test (p)	*-0.55(0.58)*	*-0.50 (0.62)*	*0 (1)*	
Men 25-64 years	1.04 (1.01-1.06)	1.03 (0.99-1.07)	1.05 (1.00-1.19)	*-0.39 (0.69)*
Women 25-64 years	0.98 (0.93-1.03)	0.97 (0.92-1.03)	1.03 (0.91-1.17)	*-0.85 (0.39)*
z-test (p)	*2.06 (0.04)*	*1.72 (0.08)*	*0.25 (0.8)*	
Men ≥65 years	1.03 (1.00-1.07)	1.03 (0.99-1.07)	1.06 (0.98-1.16)	*-0.60 (0.54)*
Women ≥65 years	1.02 (0.99-1.06)	1.02 (0.99-1.06)	1.03 (0.96-1.10)	*-0.25 (0.8)*
z-test (p)	*0.40 (0.69)*	*0.37 (0.71)*	*0.52 (0.6)*	

Table S1. Results of the sensitivity analysis using annual instead of seasonal cold spell definition, i.e. 5th percentile (threshold was -7.6°C) from the frequency distribution of daily mean temperatures over the entire study period 1.1.2000-31.12.2015, representing associations between annual cold spells and winter AMI by sex and age in Kaunas, expressed as odds ratios and 95% confidence intervals. In addition to testing differences in log odds ratios of the different subgroups within each AMI type[a], the z-test and p value were also used to test for statistically significant differences between log odds ratios of nonfatal and fatal AMI in each subgroup[b].
